# Management of obstructive sleep apnea in children: a Canada-wide survey

**DOI:** 10.1186/s40463-021-00539-5

**Published:** 2021-08-31

**Authors:** J. Cousineau, A.-S. Prévost, M.-C. Battista, M. Gervais

**Affiliations:** 1 Department of Surgery, Division of Otolaryngology – Head and Neck Surgery, Université de Sherbrooke, CIUSSSE-CHUS – 580 rue Bowen Sud, Sherbrooke, QC J1G 2E8 Canada; 2 Department of Medicine, Faculty of Medicine and Health Sciences, Université de Sherbrooke, Sherbrooke, QC Canada

**Keywords:** DISE, Endoscopy, Sleep apnea, Treatment, Diagnosis, Child, Pediatric

## Abstract

**Background:**

Obstructive sleep apnea frequently persists in children following adenotonsillectomy, which is the first-line treatment recommended for obstructive sleep apnea with adenotonsillar hypertrophy. Drug-induced sleep endoscopy (DISE) is a diagnostic tool increasingly used to assess pediatric obstructive sleep apnea, but its use has not been standardized. The overarching goal of this study was to document the current practice of Canadian otolaryngologists managing this population.

**Methods:**

A nation-wide online cross-sectional survey of Canadian otolaryngologist members of the Canadian Society of Otolaryngology – Head and Neck Surgery and the *Association d’otorhinolaryngologie et chirurgie cervico-faciale du Québec*. The 58-question electronic survey was developed based on a validated survey redaction guide with the aim to assess management and treatment of pediatric obstructive sleep apnea, as well as indications and performance of DISE. Consensus on practice items was defined by a minimum of 75% similar answers.

**Results:**

One hundred and nine Canadian otolaryngologists completed the survey on management of pediatric obstructive sleep apnea, among which 12 of them completed the questions on DISE. Overall, there was a poor rate of agreement of 55% among the respondents for the 58 questions altogether. There was a consensus to assess pediatric obstructive sleep apnea clinically ± with videos (82.6%), to assess adenotonsillar hypertrophy clinically (93.6%) and with flexible scope in the office (80.7%), as well as for the airway sites examined endoscopically during DISE. However, there was no consensus regarding anesthetic protocol and scoring system. DISE was mostly performed in cases of persistent obstructive sleep apnea after adenotonsillectomy rather than before performing any surgical procedure. There was no difference in the management of obstructive sleep apnea between otolaryngologists who perform DISE and those who do not. The only difference between otolaryngologists who practice in community centers versus in tertiary care centers was the more frequently use of the Brodsky tonsil scale by the latter ones.

**Conclusion:**

This Canadian-wide survey highlighted a lack of consensus in the management of pediatric obstructive sleep apnea and DISE. Certain aspects regarding DISE remain unclear, including establishment of its ideal timing in order to eventually avoid unnecessary tonsillectomies.

**Supplementary Information:**

The online version contains supplementary material available at 10.1186/s40463-021-00539-5.

## Background

Obstructive sleep apnea (OSA) is common in the pediatric population, with a prevalence of 1–6%. [1] When not properly treated, it can lead to physical and behavioral problems. Although different risk factors explain pediatric OSA, adenotonsillar hypertrophy is the top factor. [2] The gold standard test for OSA diagnosis is nocturnal laboratory-based polysomnography (PSG), but it is performed in less than 10% of patients prior to a surgery. [1] Although adenotonsillectomy (T&A) is the first treatment recommended by the American Academy of Pediatrics and the American Academy of Otolaryngology – Head & Neck Surgery for children with OSA and adenotonsillar hypertrophy [1, 2], the condition persists after surgery in approximately 34% of cases [3]. Failures are recorded in children with tonsils of all sizes, which means that other sites of obstruction might be responsible [4].

Awake flexible laryngoscopy, cine-MRI (Magnetic Resonance Imaging) and Drug Induced Sleep Endoscopy (DISE) are tools assessing potential sites of airway obstruction. DISE, first used for children in 1990 [5] and increasingly studied in the past few years, allows for dynamic examination of the airway during sleep. Despite a flourishing literature on the subject, there is no guidelines so far regarding DISE, making its use nonuniform among Canadian otolaryngologists. Two different rather old surveys (2004 and 2012) performed in the United States showed that pediatric otolaryngologists rarely use PSG prior to T&A and rely mostly on symptoms and physical exam to guide their management plan. [6, 7] More recently, Friedman’s and al. [8] reported a 33% rate of agreement for DISE performance among pediatric otolaryngologists practicing in tertiary care centers. Whereas most of the participants used DISE for residual OSA after T&A, respondents of the only Canadian participating institution (University of Alberta) performed DISE prior to surgery [8].

The principal objective of the present study was to assess the current practice of Canadian otolaryngologists in the management of pediatric OSA and DISE. Secondarily, we sought to analyze how the practice in tertiary care centers impacts practice and whether the practice of DISE influences the management of pediatric OSA.

## Methods

### Design

A survey was selected to answer the study question and objectives. Approval was obtained by the Research Ethics Board of the *CIUSSS de l'Estrie – CHUS.* The survey was developed using a validated guideline on the development, testing and administration of survey [9]. Important domains were identified, based on readings and clinical observations. Questions related to DISE were inspired from the 2017 Friedman survey [8], which studied DISE practice patterns in the United States only, without exploring the management of OSA. In a second group session, constituted of an otolaryngologist managing OSA and pediatric DISE, otolaryngology residents, and a methodologist, questions were reviewed and items reduced by removing redundant and less relevant questions. Dichotomous and ordinal variables were addressed in different questions and ordinal ones were addressed using a 4-point Likert scale (rarely, sometimes, often and almost always). The final survey version included 25 items addressed in 65 questions distributed in four domains: (1) Demographics; (2) Evaluation and treatment of OSA; (3) Indications of DISE; (4) DISE performance. Fifty-eight of the 65 questions specifically addressed practice patterns. A clinical scenario of a child presenting with obstructive sleep apnea was included in order to compare with information obtained in the prior sections: for several tonsil and adenoid sizes enounced, respondents were asked to indicate their management among the different options proposed.

### Testing

The survey was developed in French and back translated in English by a professional translator. The first version of the survey was administered to four otolaryngologists to detect feasibility and comprehension issues, ease of administration and assess validity. The clinical sensitivity, i.e. whether the survey specifically addresses OSA management and use of DISE and whether it covers all facets of this topic, was assessed using a clinical validated sensitivity testing tool [9] (Additional file 1). The survey was revised based on the provided feedback and its final form is provided at Additional file 2. Less than 10 min was suggested for survey completion.

### Formatting

LimeSurvey, a secure web-based and ethic-compliant platform, was selected among other available options to administer the survey. It assigned participants a unique identifier to prevent duplicate survey answer. The internet link opened on an explanation page which further detailed the study procedure and served as a consent for participation (Additional file 3).

### Participants and survey administration

An electronic mail, including an invitation letter and a link to access the survey (Additional file 4), was sent to all Canadian otolaryngologists members of the Canadian Society of Otolaryngology – Head and Neck Surgery and the *Association d’otorhinolaryngologie et chirurgie cervico-faciale du Québec* in June 2019 through these associations’ mass mail. All members were asked to complete the demographic questions, but only members who managed children with OSA where included in the statistical analyses on OSA and only members performing DISE procedures where included in the final analyses on DISE. A reminder was sent two weeks later by each association. The total collection period lasted 5 weeks (June– July 2019). The survey was completed in Canada’s both official languages, *i.e.* in either English or French. Survey completion was voluntary and answers were anonymous. E-mail addresses were kept confidential from the study investigators throughout the process.

### Sample size

Sample size was determined based on the response rates obtained from previous surveys conducted on pediatric obstructive sleep apnea [6, 7] and a reference population of 740 Canadian otolaryngologists [10]. Considering a 39% response rate, 289 members were expected to complete the survey. Although the proportion of Canadian otolaryngologists managing pediatric OSA is yet left unpublished or unreported, our team colleagues anticipated that 75% of Canadian members are involved. Accordingly, 217 members were expected to complete the survey’s question on pediatric OSA.

### Statistical analysis

All questionnaires with at least one completed question were considered. Because the study assesses Canadian current practice patterns, respondents who mentioned practicing outside of Canada were excluded. Incomplete questionnaires were included in the study. Because of some unanswered questions left by a few respondents, the denominator used for proportion calculation at each question varied. In cases of dichotomous questions, percentages of *yes* and *no* responses were computed. Ordinal values were treated as proportions. To facilitate analysis and reporting, ordinal values on the 4-point Likert scale were pooled: *rarely* and *sometimes* categories, as well as *often* and *almost always*. The proportion of each of the two groups was reported for each question.

The questions recognized as having a consensus on practice were those for which at least 75% of the participants agreed on their answer, which means a proportion, a percentage of yes or a percentage of no of 75% or above. Similarly, to conclude on an overall consensus of practice regarding pediatric OSA and DISE, a consensus in 75% or above for the 58 questions was required. The threshold of 75% was established according to publications on consensus in surgical practice [11]. For every percentage and proportions, a margin of error with a 95% confidence interval was calculated.

Subgroup analyses of how practicing at a tertiary care center influences the management of pediatric OSA and DISE use and how DISE use influences the assessment and management of pediatric OSA were pre-specified and performed. Chi-square test and Fisher’s exact tests were used. A p-value of ≤ 0.05 was considered statistically significant. Statistics were all calculated with the SPSS v25 software.

## Results

### Demographics

**Fig. 1:**
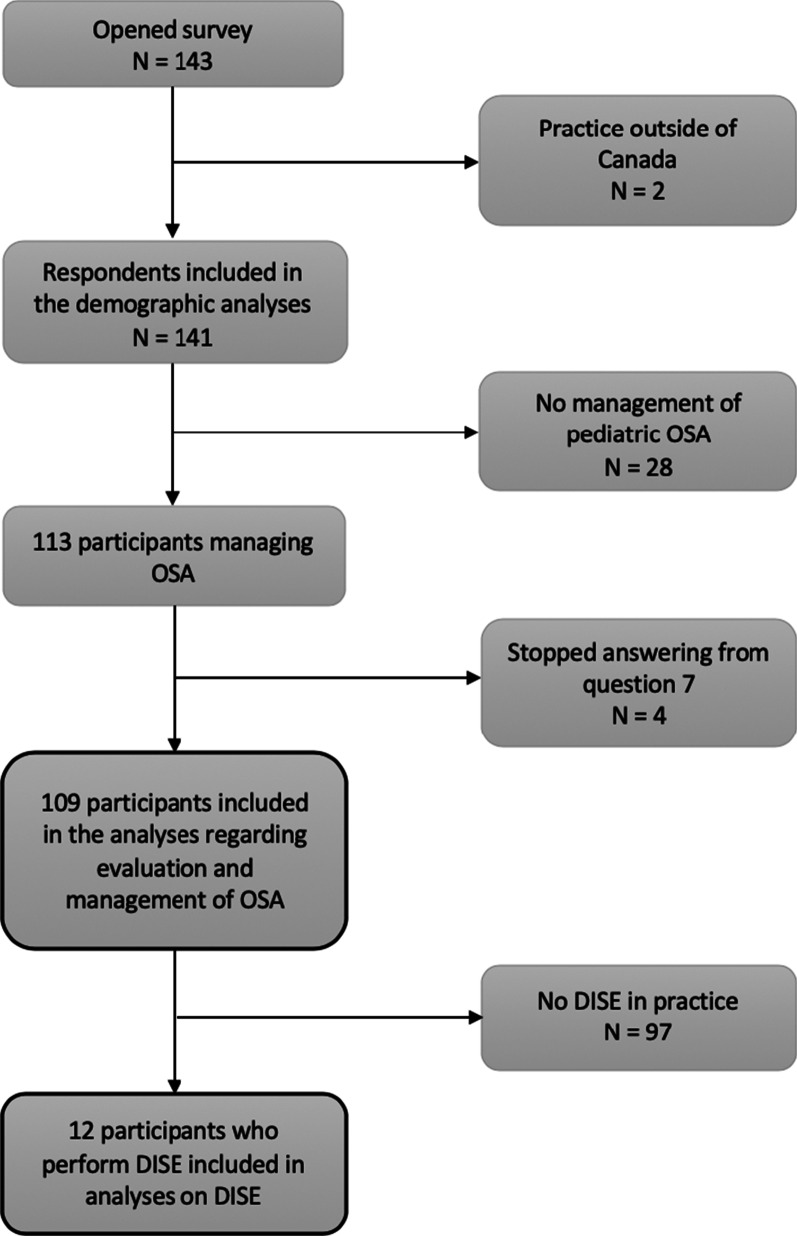
Respondents included in the statistical analsyses

One hundred and forty-three members opened the survey and completed at least one question. Two otolaryngologists practicing outside of Canada were excluded and 28 otolaryngologists not managing pediatric OSA were excluded from further analyses. Four respondents managing pediatric OSA stopped answering the questionnaire from questions related to OSA (question 7). In total, one hundred and nine participants were included in the study analysis (Fig. 1), of whom 78 practices at a tertiary care center. A total of 12 otolaryngologists, performing DISE in their practice, completed the survey. Characteristics of respondents are presented at Table 1.

**Table 1:** Characteristics of respondents (N = 109)

Characteristics	Number (%)
Language of response	
English	58 (53.2)
French	51 (46.8)
Gender	
Male	63 (57.8)
Female	46 (42.2)
Number of years in practice (mean)	13.8 (1–45)
Type of practice	
* Non-tertiary care center*	31 (28.4)
* Tertiary care center*	78 (71.6)
Nova-Scotia	4 (5.1)
Quebec	31 (39.7)
Ontario	22 (28.2)
Manitoba	2 (2.6)
Saskatchewan	3 (3.8)
Alberta	4 (5.1)
British Columbia	4 (5.1)
Unspecified	8 (10.3)

An overall agreement of 55% was observed among 32 out of the 58 questions, which is below the 75% prespecified consensus threshold. More specifically, respondents agreed on 42%, 62% and 44% of the questions regarding the management of OSA, DISE indications/performance and clinical scenario, respectively, which are all under the consensus threshold. Survey responses on consensus are presented in Table 2. Main questions with and without consensus are presented at Table 3.

**Table 2:** Responses with consensus for OSA and DISE practice survey

Question	% of yes	% of no	Response	% of response (Likert)	Consensus
**Evaluation and treatment of OSA**					
1. What do you use to objectivize OSA in your pediatric patients?		-	-	-	
c) Clinic only ± video from parents	82.6 (74.27–88.55)				Yes
8. How do you objectivize enlarged adenoids and tonsils?		-	-	-	
a) Clinically	93.6 (87.33–96.85)				Yes
c) Flexible scope in the office	80.7 (72.34–87.04)				Yes
10. In your practice, how often do children undergo a polysomnography before an adenotonsillectomy?	-	-	Likert Rarely + Sometimes	97.2 (92.22–99.07)	Yes
11. Is adenotonsillectomy the first action undertaken to treat obstructive sleep apnea in children?	75.2 (66.36–82.38)	-	-	-	Yes
**Indications regarding DISE**					
14. How often do you perform a DISE **before** an adenotonsillectomy?	-	-	Likert Rarely + Sometimes	100 (75.75–100)	Yes
15. Which of the following elements influence your decision to perform a DISE before an adenotonsillectomy?		-	-	-	
b) Nonhypertrophic tonsils	75.0 (46.77–91.1)				Yes
i) Discordance between clinical findings and severity of apnea	83.3 (55.2–95.3)				Yes
**Performing the DISE**					
19. Which anesthetic(s)/other agent(s) do you use for the DISE?	-		-	-	
a) Oral premedication		100 (75.75–100)			Yes
b) Intranasal dexmedetomidine		100 (75.75–100)			Yes
c) Nitrogen monoxide (NO) to install an IV line		91.7 (64.61–98.5)			Yes
d) Sevoflurane to install an IV line		75.0 (46.77–91.1)			Yes
g) Fentanyl		83.3 (55.2–95.3)			Yes
h) Ketamine		83.3 (55.2–95.3)			Yes
i) Topical anesthetic		100 (75.75–100)			Yes
j) Local decongestant		91.7 (64.61–98.5)			Yes
k) O2		83.3 (55.2–95.3)			Yes
21. Do you evaluate the following structures during the DISE?			-	-	
a) Nasal cavities	91.7 (64.61–98.5)				Yes
b) Adenoids/nasopharynx	100 (75.75–100)				Yes
c) Soft palate/palatine tonsils	100 (75.75–100)				Yes
d) Oropharynx	100 (75.75–100)				Yes
e) Base of the tongue	100 (75.75–100)				Yes
f) Supraglottis	100 (75.75–100)				Yes
g) Glottis	91.7 (64.61–98.5)				Yes
h) Subglottis	75.0 (46.77–91.1)				Yes
j) Bronchus		75.0 (46.77–91.1)			Yes
22. Do you use the DISE findings to perform a surgical procedure **during the same** general anesthesia session?	-	-	Likert Rarely + Sometimes	81.8 (46.77–91.11)	Yes
23. How often do you perform cine-IRM before the DISE?	-	-	Likert Rarely + Sometimes	100 (75.75–100)	Yes
**Clinical case**					
25. Patient is 7 years old, in good health and has never been operated. Snoring, daytime sleepiness and persistent difficulty concentrating in spite of a 3-month treatment with intranasal corticosteroids and montelukast. Obstructive AHI with 11 events/hour. Physical nasal exam with a speculum is normal and there is no retrognathism or high-arched palate. The cavum requested by the pediatrician is normal	-	-	-	-	-
Considering the same 7-year-old patient, what is your first therapeutic action?					
c) Adenoids < 25% and tonsils grade 3 +	T&A (81.8%)				Yes
d) Adenoids 50–75% and tonsils grade 2 +	T&A (81.8%)				Yes
g) Adenoids 50–75% and tonsils grade 3 +	T&A (100%)				Yes
h) Adenoids 50–75% and tonsils grade 4 +	T&A (100%)				Yes

**Table 3:** Questions with consensus and without consensus

Consensus	No consensus
1. OSA is objectivized clinically ± with a video from parents and enlarged adenoids and tonsils are objectivized clinically and with a flexible scope in the office	1. Role of PSG, nocturnal oximetry, questionnaire and cavum X-ray in the assessment of pediatric OSA
2. PSG is not widely used before T&A	2. The role of the Brodsky tonsil scale in the surgical decision
3. T&A is the first action undertaken for the treatment of pediatric OSA	3. The role of age and certain other patient’s characteristics in the decision to perform DISE before T&A
4. DISE is globally not performed before T&A	4. The role of DISE after an unsuccessful T&A
5. Non-hypertrophic tonsils and discordance between clinical findings and severity of apnea are elements influencing the decision to perform DISE before T&A	5. The ideal anesthetic agent and other supportive agents
6.The airway subsites assessed during DISE are the nasal cavities to the subglottis, whereas bronchi are generally not examined	6. The evaluation of the trachea during DISE performance
7. Cine-MRI is not used before DISE for assessment of airway obstruction	
8. DISE findings are not widely used to perform a surgical procedure in the same general anesthesia session	

### Evaluation and treatment of OSA

Two of the 109 participants did not perform pediatric T&A but were yet included in the analysis on management of OSA. Forty-six percent performed 1 to 5 T&A per month; 24% performed 6 to 10 T&A per month; 22.5% performed 11 to 20 T&A per month and 7.5% performed more than 20 T&A per month. Respondents agreed to rely on clinical elements and sleep videos to diagnose OSA (82.6%) and that PSG in not necessary to operate, with 76% of them who rarely request the test prior to recommending a surgery. Adenotonsillar hypertrophy was mostly objectivized clinically and with a flexible scope, with a strong consensus among respondents for clinical aspect (93.6%; 87.33–96.85). Of note, 58.7% of respondents used the cavum X-ray to assess for adenotonsillar hypertrophy, despite the availability of the flexible scope in the office. PSG was requested before adenotonsillectomy only “rarely or sometimes” by 97.2% the respondents. T&A was the first action taken by 75% of our sample to treat pediatric OSA, but there was no agreement regarding the age up to which it was their first line of treatment. There was no agreement for the use of the Brodsky tonsil scale in the decision to perform T&A.

### Indications and performance of DISE

Among the survey participants, the 12 otolaryngologists who indicated using DISE in their practice were included in the further analyses. Eleven of them performed 1 to 5 DISE per month and one of them performed 6 to 10 DISE per month. DISE was rarely (< 10% of cases) conducted before T&A for 91% (11/12) of our sample. Participants agreed on two elements that influence their decision to perform a DISE prior to a surgery: non-hypertrophic tonsils (75.0%) and a discordance between clinical findings and severity of apnea (83.3%). There was no agreement on whether to perform or not a DISE following on an unsuccessful T&A.

Most respondents performed a PSG prior to a DISE procedure “often or almost always” (7/12), but there was no clear agreement. There was no agreement regarding the optimal anesthetic agent(s) to use during DISE, however Propofol and Dexmedetomidine were the most used agents (58.3% and 50.0% of respondents used them, respectively). None of the scoring system listed in the survey was consistently used by participants and 5/12 of them (41.7%) did not use any. Consensus was noted for anatomic sites examined during DISE from nasal cavities to subglottis, whereas bronchi were not. A majority of our sample (9/12) used DISE findings to perform a surgical procedure during the same general anesthesia session in less than 50% of cases. Finally, our sample of Canadian otolaryngologists agreed on requesting “rarely” a cine-MRI before doing a DISE.

### Case scenarios

There was a strong consensus for realizing T&A when tonsils and adenoids are hypertrophied, which is what was expected for that situation (Table 4). DISE is only performed when tonsils are of lower grades; nevertheless, some respondents still consider performing T&A when tonsils are of grade 1 + or 2 + .

**Table 4:** Responses for case scenarios

	Nothing	CPAP	T&A	Adenoidectomy	Tonsillectomy	DISE
Adenoids < 25% and tonsils grade 1 +	9.1%		27.3%			63.6%
Adenoids < 25% and tonsils grade 2 +			45.5%		9.1%	45.5%
Adenoids < 25% and tonsils grade 3 +			81.8%		18.2%	
Adenoids < 25% and tonsils grade 4 +			72.7%		27.3%	
Adenoids 50–75% and tonsils grade 1 +			27.3%	54.5%		18.2%
Adenoids 50–75% and tonsils grade 2 +			81.8%			18.2%
Adenoids 50–75% and tonsils grade 3 +			100%			
Adenoids 50–75% and tonsils grade 4 +			100%			

### Pre-specified subgroup analyses

The associations between survey responses and respondents’ characteristics (type of institution of practice and if they perform DISE or not) were assessed. In comparison to ones of our sample who practice at a community hospital, those who practice at tertiary care center more frequently use the Brodsky tonsil scale (23.3% vs 50.6% respectively; P = 0.010). Respondents who perform DISE were all affiliated to a tertiary care center (P = 0.034), but there was no statistically significant difference in all the questions regarding OSA management between respondents using DISE and those who do not.

## Discussion

To our knowledge, this is the first study assessing the practice patterns of Canadian otolaryngologists in their management of pediatric OSA and use of DISE. Considering the absence of guidelines for DISE and according to a recent survey on DISE [8], we expected a low level of agreement on DISE practice, but rather predicted a better consensus on obstructive sleep apnea management, given the existence of recognized guidelines. Overall and for each domain under investigation, no clear consensus was observed.

### Evaluation and treatment of OSA

Interestingly, our results regarding the use of PSG to objectivize OSA and prior to T&A reflected the results of previous surveys, which demonstrated that PSG was obtained in 10% of children with sleep disturbance [7] and that it was requested never, rarely or sometimes in 96% of cases prior to surgery. The most recent AAO-HNS guidelines [1] on tonsillectomy recommend to perform a PSG in children with obstructive sleep-disordered breathing and one of the following: < 2 years of age, obesity, Down syndrome, craniofacial abnormalities, neuromuscular disorders, sickle cell disease, or mucopolysaccharidoses. A PSG is recommended in these patients to improve diagnostic accuracy, and to optimize perioperative planning. The responders who requested a PSG prior to T&A may or may not follow the above-mentioned criteria, but unfortunately this information was not available. Although there is no consensus regarding the use of the Brodsky tonsil scale, it is surprising to note that 30.4% of the respondents using this scale remove tonsils if graded 2 + (occupy 25–50% of the oropharynx) even if the AAO-HNS guidelines recommend tonsillectomy in children with OSA and tonsillar hypertrophy (grades 3 + or 4 +) [1]. This could be explained by the fact that the Brodsky scale might not be always accurate to predict the degree of oropharyngeal obstruction during sleep. Previous studies have shown that the Brodsky grading scale does not correlate with the OSA severity, and a significant improvement of the obstructive apnea–hypopnea index (oAHI) was found in children with grade 2 + tonsils after T&A [12, 13]. Surprisingly, even though Canadian otolaryngologists assessed sleep disordered breathing clinically, validated questionnaires of symptoms remained poorly used. In the setting of limited access to PSG, a short multi-lingual validated score such as the 6-item Severity Hierarchy Score in Pediatric OSAS [14] could be favorably used for screening and clinical decision-making. Medical treatment of OSA was not addressed in this survey, but it could have been interesting to know what treatments are used by the respondents who do not perform T&A as a first line treatment. Although tonsillectomy and/or adenoidectomy is the first line treatment recommended for children with OSA with adenotonsillar hypertrophy [1, 2], multiple trials have shown positive results with less invasive therapies, such as intranasal steroids and montelukast for nonsevere OSA [15, 16], as well as positive airway pressure as a second line treatment when surgery is contraindicated or has failed [2].

### Indications and performance of DISE

Our study confirmed that Canadian Otolaryngologists generally do not perform DISE in children who still have their tonsils. DISE in the adult population, as opposed to awake endoscopy, has been recognized to provide more clinical information on airway collapse and function. A clinical trial conducted in the adult population has demonstrated that preoperative DISE changed the surgical plan in 62% of cases. [17] However, the correlation between DISE and surgical outcome is less clear. Some conflicting data suggest that there is no benefit on surgical outcomes, [18] while other studies suggest a higher surgical success rate [19]. Although surgeries for OSA differ in adults and children, data suggest that DISE may improve surgical treatment outcomes in surgically naive children with OSA [20]. In our study, more respondents practice DISE following an unsuccessful T&A rather than prior (33% vs 0% respectively), thus suggesting a more prominent role of DISE for persistent OSA after T&A. Interestingly, in the 2017 Friedman survey [8], Canadian otolaryngologists were the only ones who routinely performed DISE prior to T&A, which is seemingly not a widespread practice pattern across the country.

There was no agreement among respondents for whether to use or not a PSG prior to performing DISE, but most respondents did not use it frequently (8/12 using it in < 50% of cases). In the 2017 Friedman survey on DISE [8], the majority of American otolaryngologists indicated requesting a PSG prior to DISE, whereas the respondents from the one Canadian institution relied on clinical elements and an overnight oximetry instead of a PSG. Our findings are thus consistent with that statement and highlight once again some limits of constraints and priorities in the Canadian health care system. This lack of accessibility to PSG emphasizes that its role needs to be better defined in this context.

There was poor agreement regarding ideal DISE anesthetic protocol, but dexmedetomidine and propofol were the two most used drugs. The absence of consensus and guidelines regarding anesthetic protocols is explained by the controversies and the limited literature regarding the effects of these drugs on the upper airway [21]. Further studies are mandated to identify the ideal agent, that would have the least incidence on airway dynamics and simulate N3 sleep phase.

The fact that 80% of respondents did not use the DISE findings to immediately proceed to a directed surgery in the same anesthesia session could be explained by different reasons; scheduling examination and treatment at different times leaves the door open for discussion and shared decision making, whereas a wide consent on different potential procedures is essential when surgery is executed immediately after DISE. Also, constraints related to time organization in the operating room may partly explain our results. Performing DISE outside of the operating room, for example in a dedicated room in the pediatric intensive care unit or an endoscopy suite, would be a potential avenue to protect operating time exclusively for surgical procedures. A recent Californian retrospective study [22] evaluated DISE procedures in two different settings, an endoscopy suite and an operating room, in terms of health care time utilization and cost outcomes. Although DISE procedures were performed on an adult population, this study showed that utilization of an endoscopy setting decreases resource utilization and financial burden, without related complications.

### Case scenarios

Surprisingly, an T&A was considered when tonsils are of grades 1 + or 2 + . The AAO-HNS guidelines for tonsillectomy recommend an T&A when there is OSA with tonsil hypertrophy, which they define as grades 3 + or 4 + [1]. Therefore, one may ask whether part of the 34% failure rate after T&A falls within the categories of children operated for small tonsils and adenoids (grades 1 + and 2 +). We think that proceeding with DISE prior to surgery in some specific cases such as in children with small adenoids and tonsils might help to give the best oriented treatment and could avoid unnecessary surgeries. A previous study showed that proceeding with DISE in surgically naive children have changed the surgical plan in 35% of the cases [23]. Moreover, as obese children are more prone to fail to improve their oAHI following a T&A, DISE could help to identify the ones that will not benefit from this surgery [2]. This could direct more rapidly these children towards non-surgical options such as continuous positive airway pressure (CPAP), weight loss, positional therapy, e.g.

### Strengths and limitations

This study builds on several strengths. To our knowledge, it is the first Canadian survey documenting the practice patterns of pediatric OSA and DISE of over 100 Canadian otolaryngologists from the country. This survey informs on areas of uncertainty and provides opportunities for improvement. Respondents were invited to answer with anonymity and without judgment or medicolegal concerns. The survey was also available in the Canada’s two official languages, thus preventing from a misinterpretation of questions. Our study also shares some limitations. The response rate was low. According to the 2016 Canadian Medical Association report [10], the estimate number of Canadian Otolaryngologists was 740. However, this number could only be speculative. The number of Canadian otolaryngologists reported by the Canadian Medical Association does not necessarily represent all members of the Canadian Society of Otolaryngology – Head and Neck Surgery and the *Association d’otorhinolaryngologie du Québec*. The number of Canadian otolaryngologists who manage pediatric OSA and who perform DISE, our population of interest, is also unavailable. An accurate response rate is therefore not accessible. Eighty percent (113/141) of participants who completed the demographic section manages pediatric OSA. If 80% of Canadian otolaryngologists manage pediatric OSA (592/740), our sample only represent 18.4% of our target population. This is significantly lower than previous surveys on DISE [6–8] and thus limits external validity. Most provinces are represented in survey responses. However, some maritime provinces are not, which may limit the generalizability. A selection bias can also emerge from the fact that several answers received may come from the same institution. As otolaryngologists within the same hospital most likely manage OSA the same way, this bias may favor larger departments or departments that have a higher participation rate. Moreover, conclusions related to DISE sections are limited due to the small sample size of included respondents practicing DISE. Again, the number of Canadian otolaryngologists performing DISE is yet unpublished, which makes it difficult to comment on the power of those analyses. As with any survey, there is potential for selection and recall bias [24] and the impact of nonresponse cannot be determined without data from non-respondents. Our study highlights answers of a sample of Canadian otolaryngologists and is therefore not generalizable to otolaryngologists practicing outside of Canada.

## Conclusion

With a global agreement of 55%, this Canadian-wide survey highlights a lack of consensus among Canadian otolaryngologists in the field of pediatric OSA and DISE in children. However, although it is the largest survey conducted in Canada so far, a larger survey or observational cohort study is mandated to further understand otolaryngologists’ practice patterns. The current practice is still not completely aligned with the American Academy of Pediatrics and the American Academy of Otolaryngology – Head & Neck Surgery clinical practice guidelines (1; 2), particularly the poor use of PSG prior to T&A and performance of tonsillectomy for relatively small tonsils. Certain elements related to DISE need further clarification, such as the role of PSG, anesthetic protocol, scoring system, timing of DISE after or prior to T&A. A prospective study would be necessary to assess the potential benefit of performing DISE before surgery in some cases, in order to prevent unnecessary T&A.

## Supplementary Information

**Additional file 1.** Clinical Sensibility Testing Tool.

**Additional file 2.** Final version of survey.

**Additional file 3.** Explanation page.

**Additional file 4.** Email Invitation Letter.

## Data Availability

The datasets analyzed during the present study are available on reasonable request via the corresponding author.

## Abbreviations

DISE: Drug induced sleep endoscopy
OSA: Obstructive sleep apnea
PSG: Polysomnography
T&A: Tonsillectomy and adenoidectomy
oSDB: Obstructive sleep disordered breathing
oAHI: Obstructive apnea–hypopnea index
