# Supplementary material for: Management of obstructive sleep apnea in children: a Canada-wide survey
Source: J Otolaryngol Head Neck Surg. 2021 Aug 31;50:53. doi: 10.1186/s40463-021-00539-5 (PMC8408936; doi:10.1186/s40463-021-00539-5)
Supplement: Supplementary file 1 — Additional file 1. Clinical Sensibility Testing Tool. [file 40463_2021_539_MOESM1_ESM.docx]

# Additional file 1 : Clinical Sensibility Testing Tool

## Survey on OSA and DISE in children

**The investigator, Dr Mireille Gervais, requests your assistance in assessing the clinical sensibility of the survey on management of obstructive sleep apnea in children and pediatric DISE by answering the following questions.**

1. To what extent are the questions directed at important issues pertaining to management of obstructive sleep apnea in children and the use of pediatric DISE among Canadian ORLs?

(Please circle your response)


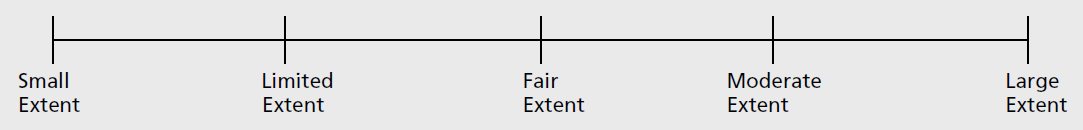


1. Are there important issues pertaining to the OSA management and the use of DISE in children that should be included in the questionnaire which have been omitted?

(Please circle your response)


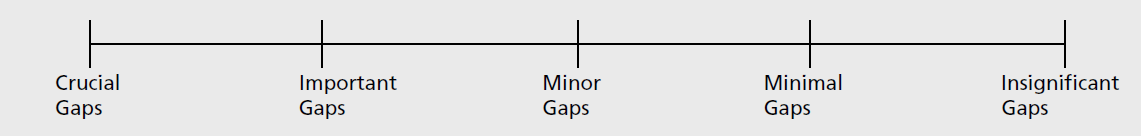


Please identify any omissions: _________________________________________________

1. To what extent are the response options provided simple and easily understood?

(Please circle your response)


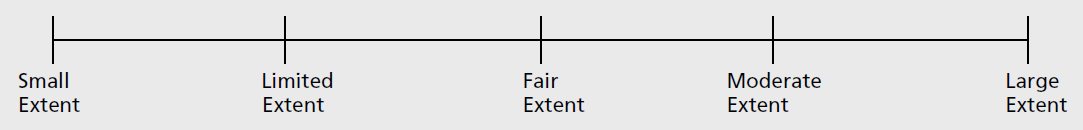


1. To what extent are questions likely to elicit information pertaining to your experience with the OSA management and the use of DISE in children?

(Please circle your response)


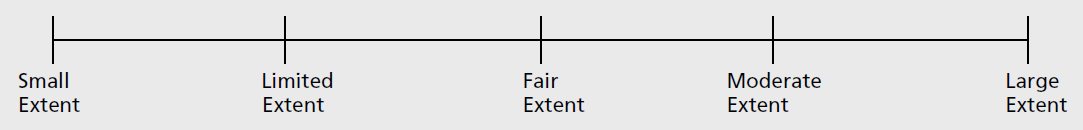


1. How many items are inappropriate or redundant?

(Please circle your response)


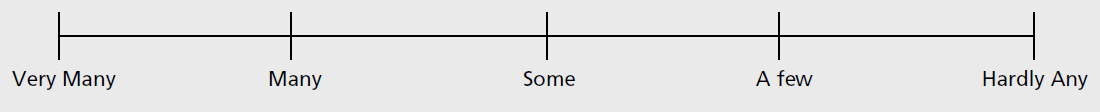


Please identify redundant or inappropriate items: _______________________________

1. How likely is the questionnaire to elicit an approximate practice level of consensus among the Canadian ORLs managing children with obstructive sleep apnea?

(Please circle your response)


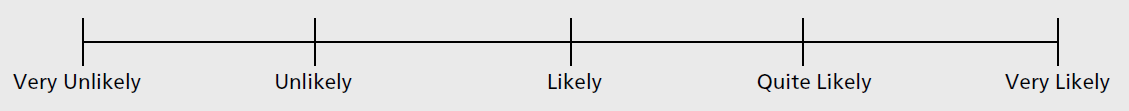


7. How long did it take you to complete the questionnaire? _________ minutes

Please feel free to provide any other feedback on the back of this form.

**Thank you for assisting us with the sensibility testing of our questionnaire!**
