# Supplementary material for: Management of obstructive sleep apnea in children: a Canada-wide survey
Source: J Otolaryngol Head Neck Surg. 2021 Aug 31;50:53. doi: 10.1186/s40463-021-00539-5 (PMC8408936; doi:10.1186/s40463-021-00539-5)
Supplement: Supplementary file 2 — Additional file 2. Final version of survey. [file 40463_2021_539_MOESM2_ESM.docx]

**Additional file 2 : Final version of survey**

**Obstructive Sleep Apnea in Children : A Canada-Wide Survey**

Demographics

1. Sex:
2. Number of years in practice:
3. Do you have a home university/institution?
4. Yes 🞏 No 🞏
5. If yes, which university/institution?
6. Do you manage children with obstructive sleep apnea?

Yes 🞏 No 🞏

🡪 If you answered NO to question 4, the survey is done. Thank you for your participation.

1. How many children with obstructive sleep apnea do you manage per month?
2. Do you perform pediatric adenotonsillectomies in your practice?

Yes 🞏 No 🞏

6a. If yes, on average, how many adenotonsillectomies do you perform per month to treat obstructive sleep apnea?

1. <1
2. 1-5
3. 5-10
4. 11-20
5. >20

Evaluation and treatment of OSA

1. What do you use to objectivize OSA in your pediatric patients?
2. Polysomnography : Yes 🞏 No 🞏
3. Nocturnal oximetry : Yes 🞏 No 🞏
4. Clinic only +/- video from parents : Yes 🞏 No 🞏
5. Validated health questionnaire : Yes 🞏 No 🞏
6. How do you objectivize enlarged adenoids and tonsils?
7. Clinically : Yes 🞏 No 🞏
8. RX cavum : Yes 🞏 No 🞏
9. Flexible scope in the office : Yes 🞏 No 🞏
10. Does the Brodsky tonsil scale influence your decision to perform an adenotonsillectomy?

Yes 🞏 No 🞏

9a. If yes, what is your threshold for removing the tonsils?

1. 1
2. 2
3. 3
4. 4
5. In your practice, how often do children undergo a polysomnography before an adenotonsillectomy?
6. Rarely (<10%)
7. Sometimes (10-50%)
8. Often (51-90%)
9. Almost always (>90%)
10. Is adenotonsillectomy the first action undertaken to treat obstructive sleep apnea in children?

Yes 🞏 No 🞏

11a. If yes, up to what age?

1. 0-5 years old
2. 6-8 years old
3. 9-11 years old
4. >12 years old

Indications regarding DISE (*Drug-induced sleep endoscopy)*

1. Do you use DISE in your practice? Yes 🞏 No 🞏

🡺If you answered NO to question 11, the survey is done. Thanks you for your participation

1. What is the average total number of DISE that you perform each month?
2. 1-5
3. 6-10
4. 11-15
5. 16-20
6. >20
7. How often do you perform a DISE **before** an adenotonsillectomy?
8. Rarely (<10%)
9. Sometimes (10-50%)
10. Often (51-90%)
11. Almost always (>90%)
12. Which of the following elements influence your decision to perform a DISE before an adenotonsillectomy?
13. Age : Yes 🞏 No 🞏
14. Nonhypertrophic tonsils : Yes 🞏 No 🞏
15. Occasional stridor : Yes 🞏 No 🞏
16. Obesity : Yes 🞏 No 🞏
17. Syndrome with craniofacial anomaly: Yes 🞏 No 🞏
18. Neuromuscular disorder/hypotonia : Yes 🞏 No 🞏
19. Comorbidities with normal weight : Yes 🞏 No 🞏
20. History of laryngomalacia : Yes 🞏 No 🞏
21. Discordance between clinical findings and severity of apnea : Yes 🞏 No 🞏
22. How often do you perform a DISE after an unsuccessful adenotonsillectomy?
23. Rarely (<10%)
24. Sometimes (10-50%)
25. Often (51-90%)
26. Almost always (>90%)
27. Do you perform a DISE only when the adenotonsillectomy is unsuccessful?

Yes 🞏 No 🞏

Performing the DISE

1. How often do you perform a polysomnography before the DISE?
2. Rarely (<10%)
3. Sometimes (10-50%)
4. Often (51-90%)
5. Almost always (>90%)
6. Which anesthetic(s)/other agent(s) do you use for the DISE?
7. Oral premedication : Yes 🞏 No 🞏
8. Intranasal dexmedetomidine : Yes 🞏 No 🞏
9. Nitrogen monoxide (NO) to install an IV line : Yes 🞏 No 🞏
10. Sevoflurane to install an IV line : Yes 🞏 No 🞏
11. Propofol : Yes 🞏 No 🞏
12. Dexmedetomidine : Yes 🞏 No 🞏
13. Fentanyl : Yes 🞏 No 🞏
14. Ketamine : Yes 🞏 No 🞏
15. Topical anesthetic : Yes 🞏 No 🞏
16. Local decongestant : Yes 🞏 No 🞏
17. O2 : Yes 🞏 No 🞏
18. I don’t know
19. Which **main** scoring system do you use most often to evaluate DISE obstruction sites?

- SERS
- VOTE
- Chan
- Boudewyns
- Fishman
- Bachar
- Other: _______________
- None

1. Do you evaluate the following structures during the DISE?
2. Nasal cavities : Yes 🞏 No 🞏

b) Adenoids/nasopharynx : Yes 🞏 No 🞏

c) Soft palate/palatine tonsils : Yes 🞏 No 🞏

d) Oropharynx : Yes 🞏 No 🞏

e) Base of the tongue : Yes 🞏 No 🞏

f) Supraglottis : Yes 🞏 No 🞏

g) Glottis : Yes 🞏 No 🞏

h) Subglottis : Yes 🞏 No 🞏

i) Trachea : Yes 🞏 No 🞏

j) Bronchus : Yes 🞏 No 🞏

1. Do you use the DISE findings to perform a surgical procedure **during the same** general anesthesia session?
2. Rarely (<10%)
3. Sometimes (10-50%)
4. Often (51-90%)
5. Almost always (>90%)
6. How often do you perform cine-IRM before the DISE?
7. Rarely (<10%)
8. Sometimes (10-50%)
9. Often (51-90%)
10. Almost always (>90%)

Clinical cases

Patient is 7 years old, in good health and has never been operated. Snoring, daytime sleepiness and persistent difficulty concentrating in spite of a 3-month treatment with intranasal corticosteroids and montelukast. Obstructive AHI with 11 events/hour. Physical nasal exam with a speculum is normal and there is no retrognathism or high-arched palate. The cavum requested by the pediatrician is normal.

1. In this case, do you perform a flexible rhino-pharyngo-laryngoscopy in the office?
2. Rarely (<10%)
3. Sometimes (10-50%)
4. Often (51-90%)
5. Almost always (>90%)
6. Considering the same 7-year-old patient, what is your first therapeutic action?

|  | None | CPAP | Adenotonsillectomy | Adenoidectomy | Tonsillectomy | DISE |
| --- | --- | --- | --- | --- | --- | --- |
| 1. Adenoids <25% and tonsils grade 1+ |  |  |  |  |  |  |
| 1. Adenoids <25% and tonsils grade 2+ |  |  |  |  |  |  |
| 1. Adenoids <25% and tonsils grade 3+ |  |  |  |  |  |  |
| 1. Adenoids <25% and tonsils grade 4+ |  |  |  |  |  |  |
| 1. Adenoids 50-75% and tonsils grade 1+ |  |  |  |  |  |  |
| 1. Adenoids 50-75% and tonsils grade 2+ |  |  |  |  |  |  |
| 1. Adenoids 50-75% and tonsils grade 3+ |  |  |  |  |  |  |
| 1. Adenoids 50-75% and tonsils grade 4+ |  |  |  |  |  |  |
