# Supplementary material for: Management of obstructive sleep apnea in children: a Canada-wide survey
Source: J Otolaryngol Head Neck Surg. 2021 Aug 31;50:53. doi: 10.1186/s40463-021-00539-5 (PMC8408936; doi:10.1186/s40463-021-00539-5)
Supplement: Supplementary file 3 — Additional file 3. Explanation page. [file 40463_2021_539_MOESM3_ESM.docx]

**Additional file 3 : Explanation page**

Project Title: Sleep Apnea in Children and Pediatric DISE: A Canada-Wide Survey

Principal Investigator: Dr. Mireille Gervais, MD, ORL and Head & Neck Surgery Department, Université de Sherbrooke

**Information**

As a Canadian otolaryngologist, you are invited to participate in the following short survey. The purpose of the survey is to assess current practices of Canadian ORLs regarding the management of children with sleep apnea and the use of pediatric sleep endoscopy.

Completing the survey will only take 10 minutes and consists of a single participation.

**Inclusion Criteria**

Questions are addressed to ORLs who manage children with obstructive sleep apnea. **HOWEVER, even if pediatric OSA is not part of your field of practice, please answer the question to this effect (#4) and complete only the first 3 demographic questions.**

**Confidentiality**

All the information gathered as part of the survey will remain confidential. Your answers to the survey will be collected via the LimeSurvey platform and will be only accessible by study investigators.

**Consent**

By completing the survey, you are giving consent for your answers to be used to publish the findings. Please note that at any time you can withdraw from participating in this survey.

**Contact**

If you have any questions or comments regarding the survey, please contact Dr. Mireille Gervais ([Mireille.Gervais@USherbrooke.ca)](mailto:Mireille.Gervais@USherbrooke.ca)), Dr. Jade Cousineau ([Jade.Cousineau@USherbrooke.ca)](mailto:Jade.Cousineau@USherbrooke.ca)), Dr. Anne-Sophie Prévost ([Anne-Sophie.Prevost@USherbrooke.ca)](mailto:Anne-Sophie.Prevost@USherbrooke.ca)) or the CIUSSS de l’Estrie-CHUS ethics committee for research (1-819-346-1110 #12856).

Your contribution is very much appreciated, **thank you** for your time.

Please note that this page can be printed out or saved in order to keep a copy of the above information.
