# Supplementary material for: Management of obstructive sleep apnea in children: a Canada-wide survey
Source: J Otolaryngol Head Neck Surg. 2021 Aug 31;50:53. doi: 10.1186/s40463-021-00539-5 (PMC8408936; doi:10.1186/s40463-021-00539-5)
Supplement: Supplementary file 4 — Additional file 4. Email Invitation Letter. [file 40463_2021_539_MOESM4_ESM.docx]

**Appendix 4 : Email Invitation Letter**

Dear Colleague,

As a Canadian otolaryngologist, you are invited to participate in the following survey about management of children with obstructive sleep apnea and the use of pediatric sleep endoscopy, inspired by a recent survey by Friedman et al.^^[[1]](#footnote-1)^^ Relating to the same field of interest, the purpose of our survey is to get an accurate picture of Canadian ORL practices for treating this pediatric pathology. The purpose is to document practices, there are no right or wrong answers.

**PLEASE NOTE: Even if your field of practice does not include pediatric sleep apnea, we invite you to answer the question to this effect (#4) and to complete only the first 3 demographic questions.**

The survey will take only 10 minutes to complete.

Your participation in this survey is crucial as you will benefit from the outcomes, considering that content from the survey will serve as the cornerstone to make changes to our practice and introduce new guidelines to current Canadian practices.

Thank you for your time, your contribution is truly appreciated!

Cordially,

Dr. Mireille Gervais, MD

Dr. Jade Cousineau, R2

Dr. Anne-Sophie Prévost, R4

ORL and Head & Neck Surgery Department

Faculty of Medicine, Université de Sherbrooke

1. Friedman NR, et al. The current state of pediatric drug-induced sleep endoscopy. Laryngoscope. 2017 Jan;127(1):266- 272 [↑](#footnote-ref-1)
